# Relation of fruit juice with adiposity and diabetes depends on how fruit juice is defined: a re-analysis of the EFSA draft scientific opinion on the tolerable upper intake level for dietary sugars

**DOI:** 10.1038/s41430-023-01258-y

**Published:** 2023-02-03

**Authors:** Victoria Chen, Tauseef A. Khan, Laura Chiavaroli, Amna Ahmed, Danielle Lee, Cyril W. C. Kendall, John L. Sievenpiper

**Affiliations:** 1 Department of Nutritional Sciences, Temerty Faculty of Medicine, University of Toronto, Toronto, ON Canada; 2 Toronto 3D Knowledge Synthesis and Clinical Trials Unit, Clinical Nutrition and Risk Factor Modification Centre, St. Michael’s Hospital, Toronto, ON Canada; 3 College of Pharmacy and Nutrition, University of Saskatchewan, Saskatoon, SK Canada; 4 Division of Endocrinology and Metabolism, Department of Medicine, St. Michael’s Hospital, Toronto, ON Canada; 5 Department of Medicine, Temerty Faculty of Medicine, University of Toronto, Toronto, ON Canada; 6 Li Ka Shing Knowledge Institute, St. Michael’s Hospital, Toronto, ON Canada

**Keywords:** Metabolic disorders, Nutrition

Low to moderate doses of 100% fruit juice have shown protective associations with cardiovascular disease [1], stroke [2, 3], stroke mortality [3], metabolic syndrome [4] and hypertension [5] in prospective cohort studies. The nutritional value of 100% fruit juice is comparable to whole fruits [6] and its consumption has been recognized as an option to meet recommendations for fruit and vegetable intake in several nutrition guidelines [7, 8]. However, the definition for 100% fruit juice is often unclear in dietary assessment questionnaires which may group together both non-defined sources of fruit juice and 100% fruit juice. These assessments may not capture the true association with 100% fruit juice, as non-defined sources of fruit juice may include fruit drinks with very little fruit juice and added sugars resembling more sugar sweetened beverages, which have shown the opposite associations with cardiovascular disease [9], type 2 diabetes [10], metabolic syndrome [11], and hypertension [12, 13] in many of the same prospective cohort studies.

The recent draft European Food Safety Authority (EFSA) assessment of the safety of dietary sugars concluded that the intake of 100% fruit juice had a positive causal relationship with type 2 diabetes and adiposity outcomes [14]. A major limitation of the EFSA assessment was that it included sources of fruit juice which did not differentiate between 100% fruit juice and non-defined sources of fruit juice. This misclassification error derives from the food frequency questionnaires used by several of the included prospective cohort studies which only asked participants questions about fruit juice and not 100% fruit juice [15–17]. Another limitation was that the EFSA assessors did not quantify the relationship between reported 100% fruit juice intake and adiposity outcomes, instead using vote counting (i.e., counting how many studies were significant versus those that were not)—an invalid approach for evidence synthesis [18].

To address the issues of misclassification of fruit juice and vote counting, we undertook a re-analysis of the prospective cohort studies identified by the EFSA assessment. We performed a quantitative meta-analysis to account for the differential weights and precision of included studies.

We stratified the included studies by the author reported definition of fruit juice into two categories. Studies that reported 100% fruit juice and pure fruit juice were categorized as 100% fruit juice. Studies that did not specify the type of fruit juice were categorized as non-specified fruit juice. We assessed the relation of the two different categories of fruit juice (100% fruit juice and non-specified fruit juice) separately with incident type 2 diabetes and adiposity outcomes (incident abdominal obesity, body weight in adults, and BMI z-scores in children). Meta-analyses were conducted by pooling beta-coefficients for continuous outcomes and log-relative risks (RRs) for incident outcomes with 95% confidence intervals (CIs) using the generic inverse variance method with DerSimonian-Laird random-effects models [19]. Heterogeneity was assessed by the Cochran Q test (significance at *P* < 0.1) and quantified by the I^2^ statistic, with I^2^ ≥ 50% and *P* < 0.1 considered evidence of substantial heterogeneity [20]. We assessed subgroup differences by fruit juice definition using the Cochrane Handbook’s recommended standard Q-test for subgroup differences (significance at *P* < 0.1) [21–23]. We used one-stage random effects meta-analysis to assess dose response for both a linear trend and a non-linear trend using restricted cubic splines with three knots [24, 25]. We tested for departure from linearity using the Wald test [26].

We included all 10 of the EFSA identified prospective cohort comparisons assessing the association of fruit juice with incident type 2 diabetes in our meta-analysis [5, 15, 16, 27–30]. There was no association of total fruit juice with incident type 2 diabetes (Fig. 1, RR: 1.08, 95% CI: 0.99–1.18) with substantial heterogeneity (I^2^ = 67%, *P*_het_ < 0.001) over a median follow-up of 12.4 years. This heterogeneity was fully explained by fruit juice definition (100% fruit juice versus non-specified fruit juice) in subgroup analyses (*P* < 0.001 for subgroup difference). 100% Fruit juice was not associated with type 2 diabetes incidence in 6 cohort comparisons (Fig. 1, RR: 0.99, 95% CI: 0.94 to 1.04, *P* = 0.64); whereas, non-specified fruit juice was associated with increased type 2 diabetes incidence in 4 cohort comparisons (Fig. 1, RR: 1.20, 95% CI: 1.13–1.28, *P* < 0.001), with no evidence of heterogeneity (I^2^ = 0%, *P*_het_ > 0.05) in either group. There was a lack of a dose-response relationship between 100% fruit juice and incident type 2 diabetes (Fig. 2a, *P* = 0.63) in the subset of 3 cohort comparisons identified by EFSA for dose-response analysis (Supplementary Table 1). In contrast, non-specified fruit juice showed a linear dose response gradient with incident type 2 diabetes (Fig. 2b, *P* < 0.001) in the subset of 4 cohort comparisons identified by EFSA for dose-response analysis (Supplementary Table 2).

**Fig. 1:**
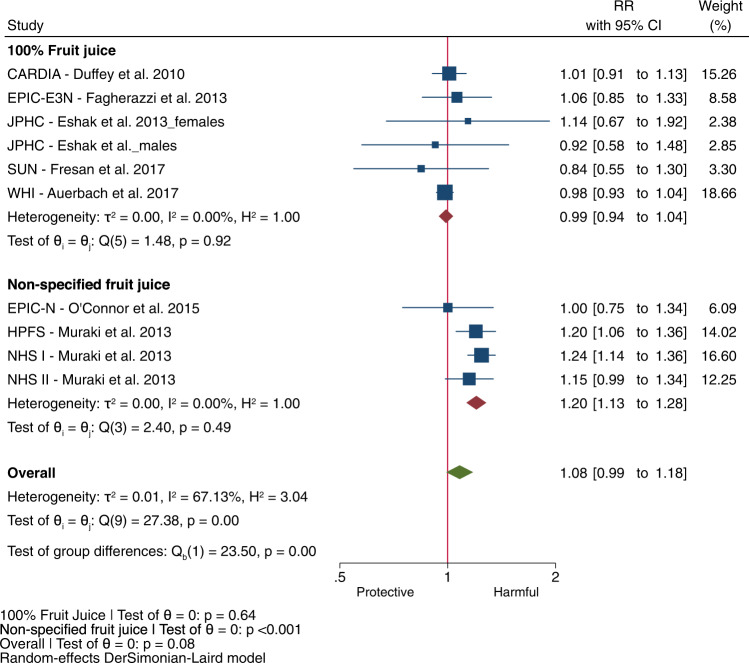
Relation of fruit juice with incident type 2 diabetes by fruit juice definition (100% fruit juice or non-specified fruit juice) for every increase in serving (250 mL) in adults in 10 prospective cohort comparisons identified by EFSA. Effect estimates for each subgroup and overall effect are represented by the diamonds. Data are expressed as relative risks with 95% confidence intervals using the generic inverse variance method with DerSimonian-Laird random-effects model. Inter-study heterogeneity was assessed using the Cochrane Q statistic and quantified using the I^2^ statistic, with significance set at *P* < 0.10 and I^2^ ≥ 50% considered to be evidence of substantial heterogeneity. Subgroup differences were tested using the standard Q-test with significance set at *P* < 0.10. CARDIA Coronary Artery Risk Development in Young Adults Study, CI confidence interval, EPIC-E3N European Prospective Investigation into Cancer and Nutrition-Etude Epidémiologique auprès des femmes de la Mutuelle Générale de l’Education National, EPIC-N European Prospective Investigation into Cancer and Nutrition-Norfolk, HPFS Health Professionals Follow-up Study, JPHC Japan Public Health Centre-based Prospective Study, NHS Nurses’ Health Study, RR relative risk, SUN Seguimiento Universidad de Navarra, WHI Women’s Health Initiative.

**Fig. 2:**
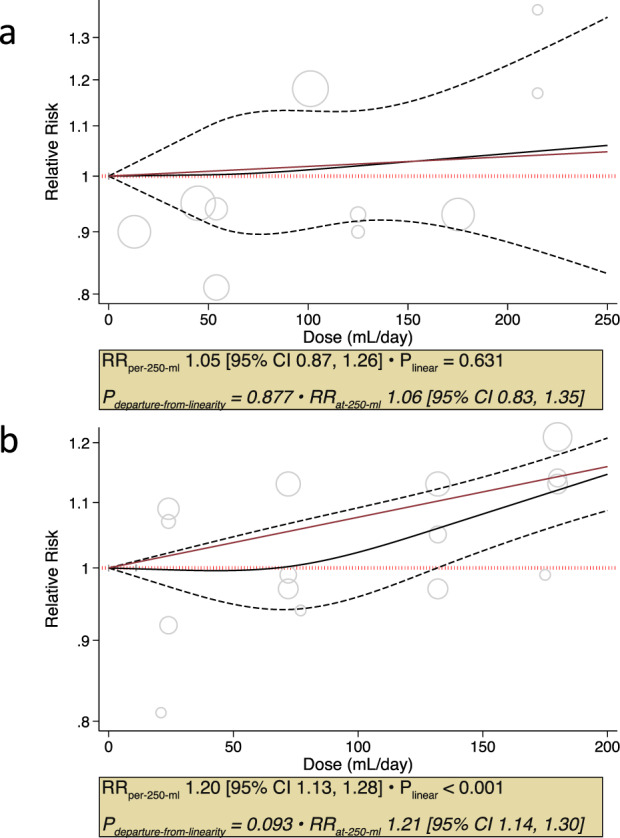
Dose response meta-analysis of 100% fruit juice and non-specified fruit juice with incident type 2 diabetes. Dose-response meta-analysis on the relation of (**a**) 100% fruit juice with incident type 2 diabetes in adults in 3 prospective cohort comparisons identified by EFSA (**b**) non-specified fruit juice with incident type 2 diabetes in 4 prospective cohort comparisons identified by EFSA. Individual comparisons are represented by the circles, with the weight of the comparison in the analysis represented by the size of the circle. The solid red line represents the linear dose response assessed by one stage linear mixed effects meta-analysis. The solid black line and outer black dashed lines represent the non-linear dose response and 95% confidence intervals, respectively, which were modelled with restricted cubic splines with 3 knots. Departure from linearity was assessed using the Wald’s test, with significance set at *P* < 0.05. CI confidence interval, RR relative risk.

The 2 prospective cohort comparisons [27, 31] identified by EFSA for the assessment of incident abdominal obesity, both of which assessed the exposure to 100% fruit juice only, showed no association of 100% fruit juice with incident abdominal obesity (Fig. 3a, RR: 0.92, 95% CI: 0.73–1.16, *P* = 0.47; moderate heterogeneity, I^2^ = 41%, *P*_het_ = 0.19) over a median follow-up of 15 years. The 4 prospective cohort comparisons [17, 32] for the assessment of change in body weight in adults showed total fruit juice was associated with an increase in body weight in adults (Fig. 3b, beta-coefficient: 0.08 kg/250 mL/yr, 95% CI: 0.06–0.10, *P* < 0.001; substantial heterogeneity, I^2^ = 79%, *P*_het_ < 0.001) over a median follow-up of 4 years with no effect modification by fruit juice definition (*P* = 0.86). Similarly, the 4 prospective cohort comparisons [33–36] for the assessment of change in BMI z-scores in children, all of which assessed the exposure to 100% fruit juice only, showed 100% fruit juice was associated with an increase in BMI z-scores (Fig. 4, beta-coefficient: 0.003, 95% CI: 0.001–0.005, *P* < 0.001; no heterogeneity, I^2^ = 0%, *P*_het_ = 0.50) over a median follow-up of 3 years, although the increases in both BMI z-scores in children and body weight in adults were clinically trivial (a change of 0.25 BMI z-score [37] and 2.5% body weight [38] or ~2 kg for 80 kg person is considered the minimally important difference in metabolic health).

**Fig. 3:**
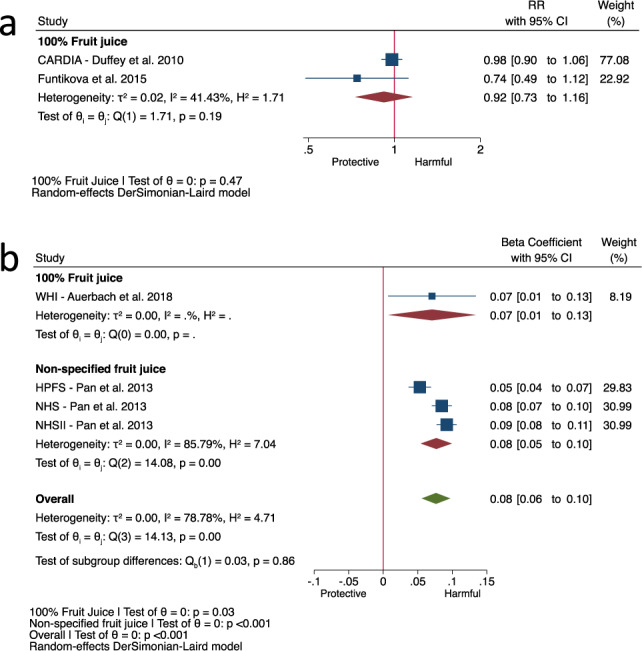
Fruit juice with incident abdominal obesity and change in body weight by fruit juice definition. Relation of 100% fruit juice with (**a**) incident abdominal obesity for every increase in serving (250 mL) in adults in 2 prospective cohort comparisons identified by EFSA and (**b**) change in body weight by fruit juice definition (100% fruit juice or non-specified fruit juice) for every increase in serving (250 mL) per year in adults in 4 prospective cohort comparisons identified by EFSA. Effect estimates for each subgroup and overall effect are represented by the diamonds. Data are expressed as relative risks or beta-coefficients with 95% confidence intervals using the generic inverse variance method with DerSimonian-Laird random-effects model. Inter-study heterogeneity was assessed using the Cochrane Q statistic and quantified using the I^2^ statistic, with significance set at *P* < 0.10 and I^2^ ≥ 50% considered to be evidence of substantial heterogeneity. Subgroup differences were tested using the standard Q-test with significance set at *P* < 0.10. CARDIA Coronary Artery Risk Development in Young Adults Study, CI confidence interval, HPFS Health Professionals Follow-up Study, NHS Nurses’ Health Study, RR relative risk, WHI Women’s Health Initiative.

**Fig. 4:**
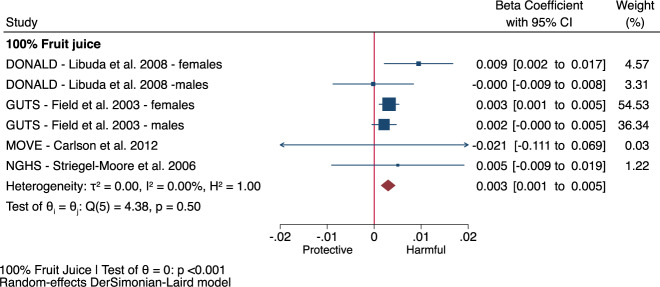
Relation of 100% fruit juice with change in BMI z-score for every increase in serving (250 mL) per year in children in 6 prospective cohort comparisons identified by EFSA. Effect estimates for each subgroup and overall effect are represented by the diamonds. Data are expressed as beta-coefficients with 95% confidence intervals using the generic inverse variance method with DerSimonian-Laird random-effects model. Inter-study heterogeneity was assessed using the Cochrane Q statistic and quantified using the I^2^ statistic, with significance set at *P* < 0.10 and I^2^ ≥ 50% considered to be evidence of substantial heterogeneity. Subgroup differences were tested using the standard Q-test with significance set at *P* < 0.10. BMI body mass index, CI confidence interval, DONALD Dortmund Nutritional and Longitudinal Designed Study, GUTS Growing Up Today Study, NGHS National Heart, Lung, and Blood Institute Growth and Health Study, RR relative risk.

Our results are in contrast with the EFSA assessment which concludes that the available evidence suggests a positive and causal relationship between the consumption of 100% fruit juice and risk of type 2 diabetes. We show that the association between total fruit juice and incident type 2 diabetes is dependent on fruit juice definition where any association with harm is only observed in studies with non-specified fruit juice. For adiposity outcomes, we show no association of 100% fruit juice and incident abdominal obesity. In prospective cohort studies which reported change in body weight (adults) or BMI z-score (children), regardless of definition of fruit juice, there was a positive association with intake of fruit juice. However, the association between 100% fruit juice and change in BMI z-score in children in our analysis was driven by one study [34] which represented 90% of the overall weight in the analysis and the increases were clinically trivial. The association was no longer significant after removal of this study (Supplementary Fig. 1).

Strengths of our reanalysis include our stratification by 100% and non-specified fruit juice, quantitative syntheses and dose-response analysis for the type 2 diabetes outcome. Our reanalysis also revealed several limitation. There was a lack of relevant data to perform dose response analyses for adiposity outcomes. Additionally, although prospective cohort studies represent the highest quality observational studies, the inability to remove unmeasured and residual confounding is inherent in all observational studies. Finally, fruit juice is generally defined as 100% fruit juice which is squeezed directly from fruit, reconstituted from concentrate [39], and labelled as such. We categorized studies in this category when they reported the intake as 100% fruit juice or pure fruit juice. Cohorts that reported only “juice” intake may have captured a wide-range of juice categories including 100% fruit juice but also those that contain little fruit juice e.g., fruit drinks, cocktails, punches, and juice beverages. The implication of this misclassification is that it is unclear exactly how much fruit juice is present in the non-defined category; therefore such studies should not be combined with those that report 100% fruit juice intake.

In conclusion, the association of fruit juice with type 2 diabetes and obesity appears dependent on the definition of fruit juice. Whereas non-specified fruit juice does show an association with increased risk of type 2 diabetes, there is no reliable association of 100% fruit juice with incident diabetes or incident abdominal obesity. More research is needed to understand the clinical importance of the small increases in body weight in adults or BMI z-scores in children associated with 100% fruit juice in the absence of adverse associations with downstream adiposity-related complications.

### Supplementary information

Supplemental Material

## Data Availability

All data generated or analyzed during this study are included in this published articles (and its supplementary information files).
