# Supplementary material for: Relation of fruit juice with adiposity and diabetes depends on how fruit juice is defined: a re-analysis of the EFSA draft scientific opinion on the tolerable upper intake level for dietary sugars
Source: Eur J Clin Nutr. 2023 Feb 3;77(7):699–704. doi: 10.1038/s41430-023-01258-y (PMC10335926; doi:10.1038/s41430-023-01258-y)
Supplement: Supplementary file 1 — Supplemental Material [file 41430_2023_1258_MOESM1_ESM.docx]

**SUPPLEMENTARY MATERIAL**

**FIGURES**

**Supplementary Figure 1.** Sensitivity Analysis with the removal of the GUTS trial (Field et al. 2003) for the relation of 100% fruit juice with change in BMI z-score

**TABLES**

**Supplementary Table 1.** Raw data for dose-response meta-analysis on the relation of 100% fruit juice and type 2 diabetes

**Supplementary Table 2.** Raw data for dose-response meta-analysis on the relation of non-defined fruit juice and type 2 diabetes

Sensitivity Analysis* with the removal of the GUTS trial (Field et al. 2003) for the relation of 100% fruit juice with change in BMI z-score

**Supplementary Figure 1.** Sensitivity Analysis* of the relation of 100% fruit juice with change in BMI z-score for every increase in serving (250mL) in children in 3 prospective cohort studies identified by EFSA. Effect estimates for each subgroup and overall effect are represented by the diamonds. Data are expressed as beta-coefficients with 95% confidence intervals using the generic inverse variance method with DerSimonian-Laird random-effects model. Inter-study heterogeneity was assessed using the Cochrane Q statistic and quantified using the I^2^ statistic, with significance set at p<0.10 and I^2^≥50% considered to be evidence of substantial heterogeneity. Subgroup differences were tested using the standard Q-test with significance set at p<0.10.

BMI, body mass index; CI, confidence interval; DONALD, Dortmund Nutritional and Longitudinal Designed Study; GUTS, Growing Up Today Study; NGHS, National Heart, Lung, and Blood Institute Growth and Health Study; RR, relative risk.

*sensitivity analysis with the removal of the GUTS trial (Field et al. 2003) which in the primary analysis carried over 90% of the weight of the analysis

**Supplementary Table 1.** Raw data for dose-response meta-analysis on the relation of 100% fruit juice and type 2 diabetes

| **Cohort** | **Participants** | **Cases** | **Reported Dose Range (mL/day)** | **Calculated Dose (mL/day)** | **Quintile** | **RR** | **LCI** | **UCI** |
| --- | --- | --- | --- | --- | --- | --- | --- | --- |
| EPIC-E3N – Fagherazzi et al. 2013 (1) | 23648 | 522 | 0 | 0 | 1 | 1 | 1 | 1 |
|  | 10617 | 200 | 0 to 26 | 12.857143 | 2 | 0.9 | 0.76 | 1.07 |
|  | 10618 | 199 | 26 to 64 | 44.857143 | 3 | 0.95 | 0.81 | 1.12 |
|  | 10618 | 246 | 64 to 138 | 101.14286 | 4 | 1.18 | 1.01 | 1.38 |
|  | 10617 | 202 | 138 to max | 175 | 5 | 0.93 | 0.78 | 1.1 |
| JPHC – Eshak et al. 2013_females (2) | 9075 | 198 | 0 | 0 | 1 | 1 | 1 | 1 |
|  | 4616 | 99 | 36 to 71 | 54 | 2 | 0.94 | 0.73 | 1.21 |
|  | 1198 | 25 | 107 to 143 | 125 | 3 | 0.9 | 0.58 | 1.4 |
|  | 559 | 18 | 179 to 250 | 215 | 4 | 1.37 | 0.79 | 2.37 |
| JPHC – Eshak et al. 2013_males (2) | 7115 | 302 | 0 | 0 | 1 | 1 | 1 | 1 |
|  | 3744 | 129 | 36 to 71 | 54 | 2 | 0.81 | 0.65 | 1.01 |
|  | 914 | 36 | 107 to 143 | 125 | 3 | 0.93 | 0.65 | 1.35 |
|  | 364 | 17 | 179 to 250 | 215 | 4 | 1.17 | 0.69 | 2 |

RR, relative risk; LCI, lower confidence interval; UCI, upper confidence interval

**Supplementary Table 2.** Raw data for dose-response meta-analysis on the relation of non-defined fruit juice and type 2 diabetes

| **Cohort** | **Participants** | **Cases** | **Reported Dose Range (mL/day)** | **Calculated Dose (mL/day)** | **Quintile** | **RR** | **LCI** | **UCI** |
| --- | --- | --- | --- | --- | --- | --- | --- | --- |
| NHS I – Muraki et al. 2013 (3) | 6605 | 921 | 0 to 22 | 11 | 1 | 1 | 1 | 1 |
|  | 5748 | 547 | 24 to 24 | 24 | 2 | 1.09 | 0.98 | 1.21 |
|  | 12170 | 1260 | 48 to 96 | 72 | 3 | 1.13 | 1.03 | 1.24 |
|  | 20706 | 1090 | 120 to 144 | 132 | 4 | 1.13 | 1.03 | 1.24 |
|  | 20876 | 2540 | 168 to max | 180 | 5 | 1.21 | 1.12 | 1.31 |
| NHS II – Muraki et al. 2013 (3) | 25120 | 672 | 0 to 22 | 11 | 1 | 1 | 1 | 1 |
|  | 16726 | 357 | 24 to 24 | 24 | 2 | 0.92 | 0.81 | 1.05 |
|  | 20259 | 777 | 48 to 96 | 72 | 3 | 0.97 | 0.86 | 1.08 |
|  | 16312 | 494 | 120 to 144 | 132 | 4 | 0.97 | 0.87 | 1.09 |
|  | 6687 | 853 | 168 to max | 180 | 5 | 1.14 | 1.02 | 1.31 |
| HPFS – Muraki et al. 2013 (3) | 3230 | 401 | 0 to 22 | 11 | 1 | 1 | 1 | 1 |
|  | 2808 | 225 | 24 to 24 | 24 | 2 | 1.07 | 0.91 | 1.26 |
|  | 6250 | 488 | 48 to 96 | 72 | 3 | 0.99 | 0.86 | 1.13 |
|  | 10682 | 460 | 120 to 144 | 132 | 4 | 1.05 | 0.92 | 1.2 |
|  | 13203 | 1113 | 168 to max | 180 | 5 | 1.13 | 1.01 | 1.27 |
| EPIC-N – O’Connor et al. 2015 (4) | 13454 | 524 | 0 | 0 | 1 | 1 | 1 | 1 |
|  | 3849 | 97 | 1 to 40 (21) | 21 | 2 | 0.81 | 0.65 | 1.01 |
|  | 3618 | 109 | 41 to 122 (77) | 77 | 3 | 0.94 | 0.76 | 1.16 |
|  | 3732 | 117 | 123 to 1372 (175) | 175 | 4 | 0.99 | 0.8 | 1.22 |

RR, relative risk; LCI, lower confidence interval; UCI, upper confidence interval

**References**

1. Fagherazzi G, Vilier A, Saes Sartorelli D, Lajous M, Balkau B, Clavel-Chapelon F. Consumption of artificially and sugar-sweetened beverages and incident type 2 diabetes in the Etude Epidemiologique aupres des femmes de la Mutuelle Generale de l'Education Nationale-European Prospective Investigation into Cancer and Nutrition cohort. Am J Clin Nutr. 2013;97(3):517-23.

2. Eshak ES, Iso H, Mizoue T, Inoue M, Noda M, Tsugane S. Soft drink, 100% fruit juice, and vegetable juice intakes and risk of diabetes mellitus. Clin Nutr. 2013;32(2):300-8.

3. Muraki I, Imamura F, Manson JE, Hu FB, Willett WC, van Dam RM, et al. Fruit consumption and risk of type 2 diabetes: results from three prospective longitudinal cohort studies. Bmj. 2013;347:f5001.

4. O'Connor L, Imamura F, Lentjes MA, Khaw KT, Wareham NJ, Forouhi NG. Prospective associations and population impact of sweet beverage intake and type 2 diabetes, and effects of substitutions with alternative beverages. Diabetologia. 2015;58(7):1474-83.
